# Physical Activity as a Treatment for Cancer-Related Fatigue in Children, Adolescents and Young Adults: A Systematic Review

**DOI:** 10.3390/children10030572

**Published:** 2023-03-17

**Authors:** Mareike Kuehn, Lena Wypyrsczyk, Sandra Stoessel, Marie A. Neu, Lisa Ploch, Elias Dreismickenbecker, Perikles Simon, Joerg Faber

**Affiliations:** 1 Center for Pediatric and Adolescent Medicine, Department of Pediatric Hematology/Oncology, University Medical Center of the Johannes Gutenberg—University Mainz, 55131 Mainz, Germany; 2 Institute of Sport Science, Department Sport Medicine, Rehabilitation and Disease Prevention, Johannes Gutenberg University, 55128 Mainz, Germany

**Keywords:** cancer-related fatigue, childhood cancer, physical activity, exercise, systematic review

## Abstract

Background: Cancer-related fatigue (CRF) is one of the most common and distressing symptoms in paediatric oncology. Based on previous studies, physical activity interventions are considered to be effective in reducing CRF in adult cancer patients. Aim: The aim of this systematic review is to investigate whether physical activity interventions can reduce CRF in paediatric patients undergoing cancer treatment. Methodology: A systematic literature search was conducted in PubMed and Sport-Discus in October 2021 to identify intervention studies examining the effects of physical activity on CRF in cancer patients ≤ 21 years of age. Their methodological quality was assessed using the JBI Critical Appraisal Tool. Results: A total of 20 studies (seven randomized-controlled, six quasi-experimental and seven single-arm intervention trials) were included in the review. Nine studies reported significant positive effects of physical activity interventions on CRF in group comparison or within groups. Eleven trials reported no significant changes in CRF. Conclusion: Physical activity as a therapeutic intervention in paediatric oncology may have the potential to reduce CRF in childhood cancer patients undergoing cancer treatment. Further high-quality studies with large samples are needed to verify these results and to assess the interdependence of dose and response of physical activity interventions.

## 1. Introduction

One of the most common and most significant side effects of cancer and cancer therapy is cancer-related fatigue (CRF) [1,2,3,4,5,6,7,8]. The multidimensional fatigue syndrome affects cancer patients of all ages regardless of the cancer entity and can occur at any time before diagnosis, during cancer treatment, but also years after treatment completion [6,7,9,10,11,12,13].

The aetiology of fatigue is multifactorial and can be attributed to the cancer disease itself, the anti-cancer treatment as well as other medical and psychological factors [14,15]. CRF manifests in particular through severe exhaustion, which cannot be counteracted even by rest and sufficient sleep [7,12,16,17]. In this context, CRF and the associated impairments can lead to an enormous reduction in the overall quality of life, especially in children, adolescents and young adults, as the patients can no longer maintain their usual lifestyle and their participation in social interaction with their peers is significantly restricted [2,18,19,20,21].

A variety of pharmacological and non-pharmacological approaches have been investigated for the treatment of CRF; nevertheless, no gold-standard therapy has been established so far [12,22]. While non-medical treatments, particularly physical activity interventions, have shown the greatest effects in reducing CRF in adult cancer patients [23,24], relatively few inconclusive studies are available concerning childhood cancer patients [25,26]. However, the beneficial results of physical activity in adult patients to reduce CRF cannot be easily transferred to children and adolescents, since the physiology and social environment in this age group differs from that of adults, and the entities of childhood cancers deviate from those in adulthood.

Due to the high need for appropriate therapeutic interventions to reduce CRF in paediatric oncology, the aim of this systematic review is to investigate whether physical activity interventions can reduce CRF in children and adolescents undergoing anti-cancer treatment.

## 2. Methods

### 2.1. Study Design

This systematic review was conducted according to the PRISMA guidelines (Preferred Reporting Items for Systematic Reviews and Meta-Analyses) [27] and registered retrospectively in the international prospective registry for systematic reviews (PROSPERO: CRD42022296879).

### 2.2. Search Strategy

A literature search was conducted in October 2021 in PubMed (MEDLINE) and SportDiscus by the two first authors and included publications that were published until 7 October 2021 in the corresponding databases.

The search strategy was developed using terms that combined the population (“oncology OR child oncology OR adolescent oncology”), the interventions (“exercise OR sport”) and the outcome of interest (“fatigue”) and was conducted using mesh terms and the Boolean operators “AND” and “OR” (see Table S1).

The results of the literature search were compiled into a bibliography using EndNote™20 from Clarivate™ and checked for duplicates.

### 2.3. Study Selection

The systematic review included peer-reviewed original articles, reviews and meta-analyses for which both abstracts and full texts were available in German or English.

Further inclusion criteria for the study selection were:

- a study population consisting of cancer patients,
- a study population in which at least 75 percent of the subjects were children, adolescents or young adults under 21 years of age,
- the conduct of the studies during cancer treatment (intensive and maintenance therapy),
- the recording of the outcome CRF,
- the implementation of a physical activity intervention, and
- the examination of the association between the physical activity intervention and CRF.

No further restrictions were made regarding the type of cancer treatment (e.g., chemotherapy, radiotherapy or immunotherapy) or the type of measurement instrument used to determine CRF. Physical activity interventions included any type of intervention in which skeletal muscles produced any bodily movement (e.g., aerobic or resistance exercises, yoga, coordination and balance exercises, walking interventions) [28]. Only the most recent version of a publication was included. Randomised controlled trials (RCTs), quasi-experimental trials (QETs) and single-group intervention studies (SGI) were eligible; non-intervention studies (e.g., cross-sectional studies) were excluded. Articles that exclusively obtained qualitative data were excluded. The study selection was performed independently by the first authors (M.K. and L.W.). The reviewers first examined the titles and abstracts of the studies and subsequently reviewed the remaining studies’ full texts according to the inclusion and exclusion criteria. In the case of contradictory results between the two reviewers regarding the selection of studies, another independent reviewer was consulted to reach a common consensus in the course of a discussion. The interrater reliability was determined by calculating Cohen’s kappa (κ). Following McHugh (2012) [29], we categorised the level of agreement for values of kappa between 0.60 and 0.79 as ‘moderate’, values between 0.80 and 0.90 as ‘strong’ and values above 0.90 as ‘almost perfect’. We interpreted a value of kappa below 0.60 as inadequate agreement between the raters [29].

As the included reviews contained studies that were partly already found in the literature search and declared as eligible for this review, only additional studies from these reviews were extracted to avoid bias in the results due to double inclusion. Therefore, an additional screening of the identified reviews was performed. Articles were included in the present systematic review when they met the inclusion criteria.

### 2.4. Methodological Quality

The methodological quality of the studies was assessed using the “Joanne Briggs Institute (JBI) Critical Appraisal Tools” for randomised controlled and quasi-experimental studies. With the help of the tools, possible biases in the study results could be determined by answering certain questions about possible deficiencies in the planning, implementation and evaluation [30].

The “JBI Critical Appraisal Tool” for randomised controlled trials assesses the methodological quality of the included studies in 13 items; the “JBI Critical Appraisal Tool” for quasi-experimental studies comprises nine items [30].

Studies were ranked according to their score as follows: high quality (10 or 7 points or more), moderate quality (5–9 or 4–6 points) and low quality (4 or 3 points or less). The classification was defined by the authors and is not validated.

Methodological quality was assessed by two independent reviewers (M.K. and L.W.) (see Table S2). In the case of discrepancies in the assessment of study quality, another reviewer was consulted. No studies were excluded due to their quality.

### 2.5. Data Extraction and Analysis

The relevant data of the included studies were extracted independently by two reviewers (L.W. and M.K.) into a data collection form created for this purpose.

The following data were extracted:

- Reference (author, year)
- Country
- Study design
- Type of physical activity intervention
- Study population (intervention and control group (number, age))
- Entity
- Therapy
- Description of the intervention (duration, frequency, duration of a session, intensity)
- Organisation and setting
- Measurement of CRF and measurement time points
- Results of the interventions on CRF (scores) and other outcomes (e.g., health-related quality of life)
- Methodological quality.

The training intensity was classified according to the guidelines of the American College of Sports Medicine (ACSM) and was subdivided into three categories: “low”, “moderate” and “vigorous”. In case the included studies did not mention the training intensity applied during the intervention, but provided information about the maximum heart rate in aerobic exercises or 1-RM for strength exercises, which are used to define the intensity of physical exercises, the ACSM guidelines were consulted. For endurance training, the intensity was determined by the maximum heart rate. If a heart rate of less than 64% of the maximum heart rate was reached in a training session, this corresponded to a low training intensity. A moderate intensity was achieved at 64 to 76% of the maximum heart rate. At a heart rate above 76% of the patient’s maximum heart rate, this corresponded to vigorous-intensity training. For strength training, the intensity was determined using the one-repetition maximum (1-RM). A strength training that was carried out at less than 50% 1-RM had a low intensity. A moderate intensity corresponded to 50 to <70% 1-RM. Vigorous-intensity training was 70% 1-RM or above [31].

The extracted data from both authors were reviewed and verified for completeness. In the case of discrepancies, consensus was reached through discussion.

The results of the included studies regarding the outcome fatigue were reported descriptively. We summarised the main characteristics and results of each study in tables to facilitate the identification of patterns in the collected data and to present the results in a concise synthesis. Due to the heterogeneity of the studies, no meta-analysis was performed. In order to assess the certainty of the findings, the GRADE working group (Grading of Recommendations, Assessment, Development, and Evaluations) was referred to [32].

## 3. Results

### 3.1. Study Selection

The systematic literature search in PubMed (MEDLINE) and SportDiscus yielded a total of 303 articles. After removing duplicates, 292 articles remained, which were examined in the abstract screening. Cohen’s kappa was κ = 0.64.

Eighty-nine articles remained for which the reviewers re-examined the full texts according to the inclusion and exclusion criteria. Discrepancies in the reviewers’ ratings (M.K. and L.W.) occurred in only two of the full study texts. However, consensus was reached through discussion and the involvement of the third reviewer. The interrater reliability was accordingly almost perfect with a Cohen’s kappa of κ = 0.95.

Twenty-five full-text articles were found, thereof 16 original articles and nine reviews. The latter were then excluded. Nevertheless, an additional screening of those nine reviews was performed resulting in the identification of four additional original articles. Thus, a total of 20 original studies that investigated the effect of physical activity interventions on CRF in the relevant age group were included. The reviews were not further included in the analysis. Of the primary studies extracted from the reviews, seven duplicates were removed. Subsequently, the nine remaining primary studies were also screened for the inclusion and exclusion criteria resulting in four additional articles being included in the review.

**Figure 1:**
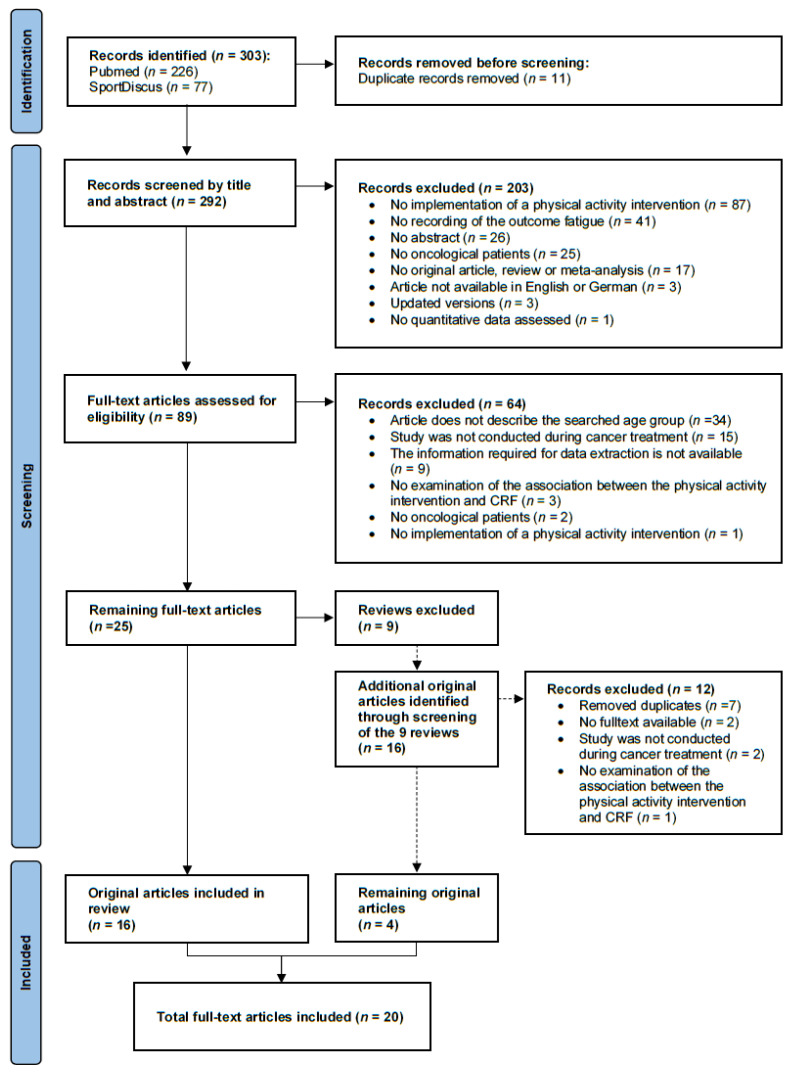
PRISMA flowchart of the study selection.

In total, 20 randomised controlled and quasi-experimental studies were included in the systematic review. Figure 1 shows the study selection process.

### 3.2. Study Characteristics

The 20 included studies contained seven randomised controlled trials (RCTs) [33,34,35,36,37,38,39]. The other included studies were six controlled quasi-experimental studies [40,41,42,43,44,45] and seven prospective single-group intervention studies [46,47,48,49,50,51,52].

The interventions were mainly conducted in Europe: four of the studies took place in Germany and one each in Italy, Finland and the Netherlands. Four studies were conducted in the USA and two in Canada. Two studies were conducted in Australia and another two in Taiwan. One study each was realised in China, India and Indonesia. All articles were published between 2007 and 2021.

The studies were generally conducted to investigate both the feasibility and the effects of a physical activity intervention on physical and psychological outcomes in childhood cancer patients.

The most frequently used measurement instrument for CRF was the Pediatric Quality of Life Inventory™ Multidimensional Fatigue Scale (PedsQL MFS), which was used in 13 studies. The items of the PedsQL MFS assess general CRF, cognitive CRF, sleep and rest. In addition, the different versions of the “Fatigue Scale” for children, adolescents, their parents and staff (FS-C, -A, -P, -S) were used in six studies. The Piper Fatigue Scale and the Childhood Fatigue Scale (CFS) were used to assess CRF in one study each.

In addition to CRF, the majority of studies assessed other outcomes such as HRQOL (*n* = 12), physical activity (*n* = 9), sedentary behaviour (*n* = 1), motor performance (endurance, strength, mobility, etc.) (*n* = 8), sleep quality (*n* = 3), various anthropometric data (*n* = 2) and emotional and psychosocial outcomes such as mood, depression, self-efficacy and behavioural problems (*n* = 3). Table 1 reflects the characteristics of the included studies.

#### 3.2.1. Study Participants and Control Group

A total of 660 participants were included in the systematic review, with sample sizes varying from nine [51] to 70 participants [37].

The age of the subjects ranged from three to 25 years. Eight studies did not specify the age range of the patients included in their study. Overall, the population consisted of 41% female and 59% male cancer patients.

**Figure 2:**
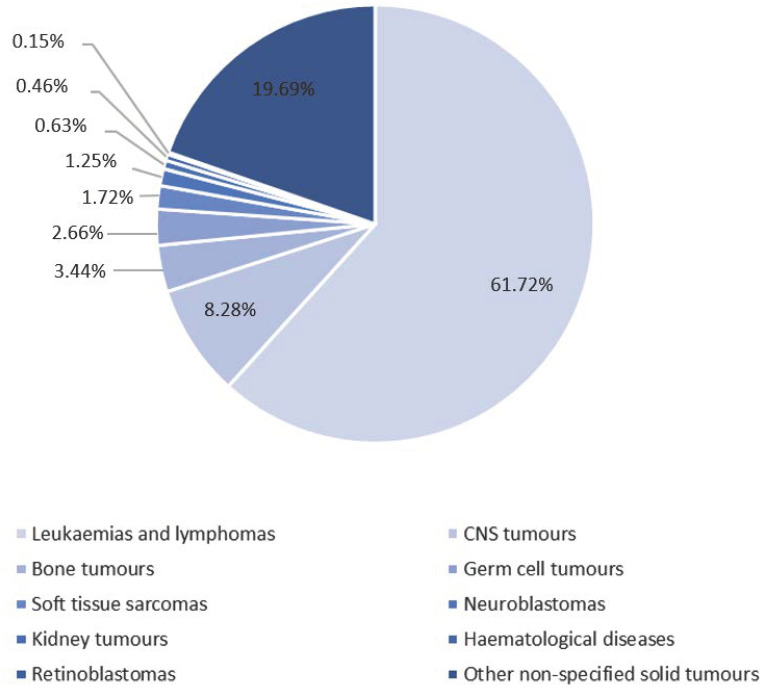
Investigated entities of the study participants (*n* = 640) of the included studies.

The tumour entities examined in the included studies covered the entire spectrum of childhood cancer, with leukaemia being the most frequently examined entity. An overview of the entities of the subjects examined in the included studies are shown in Figure 2. Half of the studies compared the intervention group with a control group, i.e., with patients receiving standard care. In the study by Hamari et al. (2019) [35], the control group additionally received a physical activity recommendation, but no supervised physical activity intervention. The control group of the quasi-experimental trial by Däggelmann et al. (2017) [40] compared the intervention group with the healthy siblings of the patients, who received the same intervention as the patients. In the study by Hooke et al. (2019) [41], a historical control group was included for the group comparison.

Further information regarding the study participants is presented in Table 1 and Table 2. 

#### 3.2.2. Physical Activity Interventions

The variety of physical activity interventions in the 20 included studies comprised endurance and strength training as well as exercises to promote coordination, balance and flexibility, walking interventions with fitness trackers, technology-based physical activity interventions, but also yoga and specific coaching to increase physical activity.

Three of the included studies performed only endurance training [34,36,42] and two studies performed a combination of endurance and strength training [33,39]. Seven other studies performed multimodal training consisting of endurance, strength, flexibility and coordination training, whereby the endurance and strength components made up the majority of the training. In two of these studies, relaxation techniques were additionally carried out [37,44].

Three studies conducted technology-based physical activity interventions using gaming consoles or a video training [35,45,51], and three other studies used walking interventions with fitness trackers [48,50,52]. Moreover, one study used yoga [47] and one study used coaching to increase physical activity [41].

In addition to the physical activity intervention, several of the included studies implemented an educational intervention [37,49,50], psychosocial training [39] or psychological, occupational therapy and experiential education [40].

**Figure 3:**
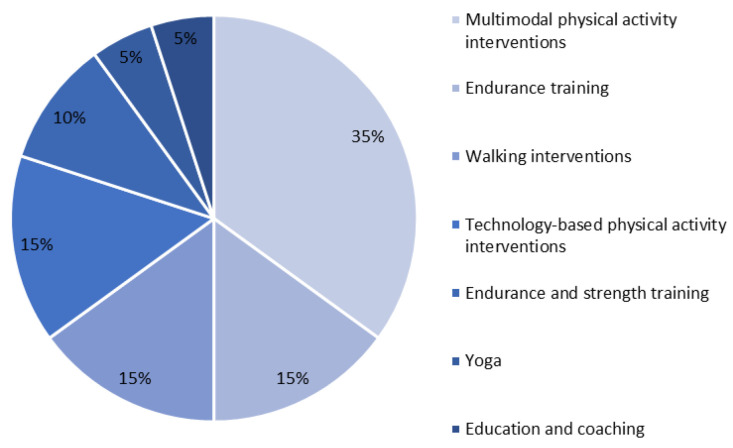
Types of training implemented in included studies (*n* = 20).

The types of training used in the included studies are shown in Figure 3.

#### 3.2.3. Implementation of the Physical Activity Interventions

Twelve studies conducted a supervised physical activity intervention, in four studies the subjects trained partially supervised and in another four studies, the participants did not receive supervised training sessions.

The physical activity interventions lasted two days to six months and were conducted at least once a week. However, the overall frequency of physical activity sessions ranged up to a maximum of ten physical activity sessions per week, with twice-daily sessions five days per week.

The duration of a training session varied between 10 and 90 min, with most studies reporting a training duration of 45 or 60 min.

In only eight studies the intensity of the physical activity interventions was described and varied between low and vigorous intensity. On average, the training intensity of the different physical activity interventions was in the moderate range.

### 3.3. Methodological Quality

The methodological quality of the RCTs was rated on average as moderate with 7.6 out of a possible 13 points (58.46%).

The methodological quality of the quasi-experimental controlled trials and single-group intervention studies was also rated as moderate with an average of 4.8 out of a possible nine points (53.33%) (see Table S3).

In only one study the methodological quality was rated low [46], but to provide a comprehensive insight of potentially promising results on exercise and CRF in childhood cancer patients, this study was also included.

Table 3 and Table 4 depict the results of the methodological quality assessment of the included randomised-controlled trials, quasi-experimental studies and single-group intervention studies.

### 3.4. Effect of Physical Activity Interventions on CRF

Eight of the 20 studies found significant reductions in fatigue in the intervention group after the physical activity intervention [34,37,38,40,49,50,51,52]. In studies in which significant reductions in CRF occurred, the percentage change could be obtained from seven studies. In these, fatigue was reduced by an average of 13.54% (range 2.0–26.8%). Nine studies reported no significant changes in CRF in the examined patients [35,46,48]. Three studies did not examine the effect of the intervention within the intervention group when comparing the fatigue level before and after the intervention, but only assessed group differences of the intervention and control group [36,42,44]. Studies comparing the intervention group with a control group showed a significant benefit for the intervention group in three studies [37,38,42]. In six studies, CRF did not differ significantly between the groups directly after the intervention [33,35,36,39,44,45].

In the study by Hooke et al. (2019) [41], a comparison between the intervention group and the historical control group only took place during the intervention period. During the intervention, no significant difference was found between the two groups in terms of fatigue.

In one study fatigue was significantly higher in the intervention group compared to a relaxation group following the intervention phase [34].

In the study by Däggelmann et al. (2017) [40] no comparison of CRF values between the intervention and the control group, which consisted of healthy siblings, was performed after the intervention. Rosenhagen et al. (2011) [43] did not compare the extent of CRF after the intervention with that in the historical control group.

Eight of the included studies performed follow-up examinations. In four of these studies [33,35,39] no differences were observed when comparing CRF at baseline, directly after the intervention or during follow-up.

Three studies showed significant improvements in CRF following the intervention phase that were maintained in the follow-up examinations [37,39,40,50] and another study showed further significant improvements in CRF [45,49]. Three of the included studies also revealed relevant correlations between different factors assessed in the studies and the main outcome CRF. A positive correlation according to CRF was indicated when examining the sleep quality of the patients [36]. Further correlations could be established in relation to physical activity. It was shown that increased physical activity is associated with better physical and mental well-being, increased self-esteem and reduced CRF [36,48].

Four of the studies with significant positive effects on CRF used multimodal physical activity interventions consisting of endurance, strength, balance and flexibility training, two studies applied solely endurance training [34,42] or a pedometer-based walking intervention [50] and one study implemented a technology-based physical activity intervention using a game console [51].

Significant positive effects were particularly found in supervised studies (*n* = 5). Three studies with partially supervised training also demonstrated significant positive effects on CRF. Table 5 summarizes the overall results of the investigated studies.

The certainty of the results was very low according to GRADE. Safety was downgraded due to a risk of bias, inconsistency in reported study results and insufficient precision due to small subject numbers.

## 4. Discussion

The aim of this systematic review was to examine whether physical activity interventions can reduce CRF in children and adolescents undergoing anti-cancer treatment. A total of 20 studies was identified that investigated the effects of physical activity interventions in children, adolescents and young adults on CRF.

The results indicate that physical activity interventions may have the potential to reduce CRF in children, adolescents and young adults with cancer: Significant positive effects of the different interventions on CRF were shown in almost half (*n* = 9) of the 20 investigated studies. However, due to missing [49,50,51,52] or non-comparable [40] control groups, it is not possible to say with certainty whether the improvements are due to the performed physical activity interventions or to other causes. Nine studies reported no significant changes in CRF in the patients studied. The small sample sizes, but also the methodological limitations of the studies, may have meant that some studies did not find significant changes. This would mean that these studies are underpowered.

In the studies that additionally compared the outcome CRF in the intervention and control group after the physical activity intervention, three trials found a significant advantage for the intervention group. As these are randomised controlled trials of comparatively high quality with large sample sizes, these results are considered particularly relevant to answering the research question of this review. However, this was contradicted by four other studies [33,35,36,39,44,45] with comparatively large samples and relatively high quality, which did not find any significant differences between the groups. Due to this controversy, it is not yet possible to clearly state to what extent physical activity interventions can reduce CRF in childhood cancer patients. Only one study observed a significant disadvantage for the intervention group regarding CRF after the physical activity intervention [34]. However, in this study the intervention group was not compared to the standard care, but to a group that received relaxation techniques. Therefore, according to current studies, it cannot be assumed that physical activity in childhood cancer patients increases CRF, but rather leads to an improvement in fatigue symptoms. In addition, the possible potential of relaxation techniques to reduce CRF compared with physical activity interventions should be investigated in further studies.

Of the eight trials with follow-up, three trials showed a significant reduction in CRF compared with pre-intervention, both immediately post-intervention and at follow-up. In one trial, a further reduction in CRF was observed at follow-up. However, due to the lack of comparable control groups, it is currently not possible to determine whether the persistence or further improvement in CRF is due to a natural decline in fatigue at the end of cancer treatment or to long-term effects of the physical activity intervention. Possible long-term effects of physical activity on CRF should therefore be further investigated in the future.

Furthermore, the examination of the included studies within the scope of the review enabled statements to be made about the types of training that are already being used in paediatric oncology as part of targeted physical activity interventions to reduce CRF.

All included studies integrated some form of endurance training into their interventions, and most significant positive effects on CRF were found in studies that combined strength, flexibility and coordination training with endurance training [37,38,40,49]. Thus, the results are in line with current meta-analyses of adult cancer patients with different tumour entities, which were able to show significant improvements in CRF with low to moderate effect sizes for both endurance or strength training and the combination of different types of training [23,24,53,54].

However, due to the heterogeneity in the reviewed studies, no conclusions can currently be made about which type, duration, frequency and intensity of physical activity is most effective in reducing fatigue. The different results regarding the effectiveness of exercising while adhering to the individual dosage of training are also strongly discussed in the context of current scientific research in adult cancer patients.

For example, the effects of exercise therapy and various physical activity interventions may have a certain dose-dependency, whereas some researchers report that the type of physical activity and strict adherence to certain exercise norms are negligible for the treatment of CRF [12,55]. According to the studies by Thong et al. (2020) [12], there are as well very diverse results on the effectiveness of different types of training. The study by Mustian et al. (2017) [24], for example, showed that there was no direct correlation between the type of training and effectiveness. In contrast, Hilfiker et al. (2018) [56] and Patel and Bhise (2017) [57] reported that endurance training in particular is very effective in CRF therapy.

Tian et al. (2016) [58] additionally investigated whether the type of endurance training, such as cycling or walking, had different effects on patients’ CRF. However, they did not find any significant differences, so that the general positive effect of endurance training can be assumed. Furthermore, Tian et al. (2016) [58] found that the training frequency as well as the duration of a training session can influence the effectiveness of the intervention. In addition, van Vulpen et al. (2020) [59] showed that interventions lasting twelve weeks or less seem to be particularly effective in the treatment of CRF.

Most of the physical activity interventions considered in this review were supervised or at least partially supervised, so eight of the studies with significant positive effects on CRF included supervised (five studies) or partially supervised (three studies) training. Given that previous research has demonstrated that supervised training has greater effects on both physical and psychosocial outcomes compared to non-supervised training [60,61], the focus should remain on supervised training in the future. However, it is not yet clear whether the strong effects of supervised training are due to a higher dose of high-quality training or to psychosocial effects such as increased motivation or attention [60].

In general, in interventions showing significant increases in endurance capacity [38,50,52], strength [37,38,49,51], mobility [49], general physical condition [51], quality of life and other psychological components [49,51] or sleep quality [42], CRF decreased significantly. In addition, several studies found a significant correlation between higher physical activity and lower CRF [38,48] and a significant correlation between better sleep quality and lower CRF [36]. However, based on the current studies, no conclusions can yet be drawn about the interaction mechanisms by which an increase in physical capacity might lead to a reduction in CRF. Future studies should further investigate these relationships.

The reduction in CRF in childhood cancer patients may therefore also be attributed to the effects of improved physical performance [62], positive psychological effects [63] and improved sleep quality [64], which have already been widely discussed in adult patients. Hence, further studies are needed to understand the exact mechanisms behind the improvement of fatigue through physical activity.

A limitation of this work is that although a systematic literature search was conducted, relevant studies may not have been identified. On the one hand, the literature search was only carried out in two databases, and on the other hand, not all bibliographies of the reviewed studies were checked for further relevant articles, and no review of the grey literature was carried out.

Furthermore, a meta-analysis could not be performed in this review due to some limitations of the included studies. Limitations of the studies in this context included both the study samples and the physical activity interventions used.

The samples showed a high degree of heterogeneity in terms of the age of the patients included, with studies involving only children, studies comprising adolescents and young adults and studies including all three age groups.

There was also much heterogeneity in terms of cancer diagnosis and treatment. While some trials only examined patients with leukaemia or CNS tumours, other trials investigated patients from the whole spectrum of childhood cancer diagnoses. The latter resulted in participants being studied who received completely different treatment approaches or regimens, with patients being treated with chemotherapy alone or with a combination of chemotherapy and radiotherapy. Some trials also included patients who received high-intensity stem cell transplants or underwent certain types of surgery, which led to a huge heterogeneity in the samples studied.

Moreover, the timing of the interventions varied widely between the included trials, with some interventions taking place only during the intensive cancer treatment, and others taking place not only during the intensive treatment, but also during maintenance treatment and up to the start of aftercare.

In addition, the physical activity interventions used in the trials varied considerably, which severely limits the comparability of the trials. For example, the duration of the interventions varied from two to four days to six months. In addition, some trials used daily or twice-daily sessions, while others only reported once-weekly sessions. There was even more variation in the length of the training sessions, which ranged from 15 or 30 min to 60 min per session. In addition to the limitations of the included studies mentioned above, which limited the ability to conduct a meta-analysis, further limitations of the studies reviewed need to be mentioned. A general problem is the small sample size of paediatric oncology studies, which is due to the rarity of childhood and adolescent cancers. The study results can therefore only be generalised to a limited extent. In order to investigate larger sample sizes and thus be able to generate more meaningful and generalizable results, future multicentre studies are needed.

Furthermore, in several studies, additional interventions were carried out such as educational interventions [37,49,50], relaxation techniques [37,44], psychosocial training [39] or occupational therapy [40]. Since some of these studies were able to achieve significant positive effects with regard to the outcome CRF, it remains unclear whether the positive effects are due to the physical activity intervention or the additional therapy. In order to be able to make statements about whether a physical activity intervention alone or in combination with another intervention is most effective in reducing CRF, further randomised controlled trials are needed.

## 5. Conclusions

Physical activity interventions show great effects on CRF in adult cancer patients. Therefore, the aim of the present review was to provide an overview of the results of physical activity interventions in childhood cancer patients and to discuss physical activity as a promising therapeutic intervention for CRF in the literature.

Mixed results were found in the present review regarding the effectiveness of different physical activity interventions on CRF in children, adolescents and young adults suffering from cancer, with almost half of the studies supporting the use of these measures to reduce the manifestation of CRF. Most of the studies with significant positive effects on CRF used multimodal physical activity interventions consisting of endurance, strength, balance and flexibility training and performed the training sessions (partially) supervised.

Nevertheless, further high-quality randomised controlled trials are needed to prove the effectiveness of physical activity in reducing CRF. To achieve high sample sizes and a high degree of generalisability, multicentre studies should be aimed for. In addition, further studies are urgently needed on what type and dosage of physical activity is most effective in decreasing CRF.

In conclusion, this may finally help to implement personalised exercise therapy as an evidence-based standard in clinical care for children, adolescents and young adults undergoing anti-cancer treatment.

## Figures and Tables

**Table 1:** Characteristics of included studies.

Author (Year)	Study Design	Population	Cancer Entity	Physical Activity Intervention	Control Group
Atkinson et al. (2021) [33]	RCT	**N = 43** Maintenance therapy After care *IG (n = *22*):* age (mean): 20.59 ± 3.2 y *CG (n = *21*):* age (mean): 20.9 ± 2.6 y	Lymphomas 58.1% Germ cell tumours 18.6% Leukaemia 9.3% Bone tumours 7.0% Soft tissue sarcomas 4.7% CNS tumours 2.3%	**Endurance and strength training (supervised)** *Duration of intervention:* 10 Weeks *Frequency:* 2×/Week *Duration of a session:* 15–30 min *Intensity:* moderate–vigorous	Usual care
Bhatt et al. (2013) [34]	RCT	**N = 36** age (range): 8–16 y Intensive cancer treatment	Leukaemia	**Endurance training (supervised)** *Duration of intervention:* 3 Weeks *Frequency:* 5×/Week *Duration of a session:* 20 min *Intensity:* n.a.	Relaxation technique
Bogg et al. (2015) [46]	SGI	**N = 14** age (mean): 11.0 y Intensive cancer treatment	Leukaemia 36.4% Haematol. disorders 14.3% Lymphomas 7.1%	**Multimodal training (supervised)** *Duration of intervention:* Isolation phase during SCT *Frequency:* 5×/Week *Duration of a session:* 10–60 min *Intensity:* low–moderate	No CG
Däggelmann et al. (2017) [40]	QET	**N = 42** Maintenance therapy After care *IG (n = *22*):* age (mean): 10.36 ± 4.04 y *CG (n = *20*):* age (mean): 9.10 ± 5.83 y	Leukaemia 36.3% CNS tumours 22.6% Lymphomas 13.5% Neuroblastomas 4.6% Retinoblastomas 4.6% Bone tumours 4.6% Soft tissue sarcomas 4.6% Germ cell tumours 4.6% Other solid tumours 4.6%	**Rehabilitation program** **(multimodal; supervised)** *Duration of intervention:* 4 Weeks *Frequency:* n.a. *Duration of a session:* n.a. *Intensity:* n.a.	Healthy siblings receive the same intervention as the IG
Diorio et al. (2015) [47]	SGI	**N = 11** age (Median): 14.0 y Intensive cancer treatment	Leukaemia and lymphomas 72.7% Solid tumours 9.1% CNS tumours 9.1% Haematol. disorders 9.1%	**Yoga (supervised)** *Duration of intervention:* 3 Weeks *Frequency:* 3×/Week *Duration of a session:* n.a. *Intensity:* low–vigorous	No CG
Hamari et al. (2019) [35]	RCT	**N = 36** *IG (n = *17*):* age (mean): 7.8 y *CG (n = *19*):* age (mean): 7.9 y	Leukaemia 47.2% Lymphomas 30.6% Nephroblastomas 5.6% Other solid tumours 16.7%	**Technology-based physical activity intervention** **(partly supervised)** Duration of intervention: 8 Weeks Frequency: daily Duration of a session: mind. 30 min Intensity: n.a.	Written advice for PA of 30 min/ day
Hinds et al. (2007) [36]	RCT	**N = 29** *IG (n = *14*):* age (mean): 13.08 ± 2.55 y *CG (n = *15*):* age (mean): 11.92 ± 3.24 y.	Solid tumours 86.0% Leukaemia 14.0%	**Endurance training (supervised)** *Duration of intervention:* 2–4 Days *Frequency:* 2×/Tag *Duration of a session:* 30 min *Intensity:* n.a.	Usual care
Hooke et al. (2016) [48]	SGI	**N = 16** age (mean): 8.69 ± 3.09 y	Leukaemia	**Walking intervention with fitness tracker (non-supervised)** *Duration of intervention:* 2 Weeks *Frequency:* n.a. *Duration of a session:* n.a. *Intensity:* n.a.	No CG
Hooke et al. (2019) [41]	QET	**N = 57** *IG (n = *30*):* age (mean): 12.0 ± 3.6 y *CG (n = *27*):* age (mean): 12.8 ± 3.3 y	Leukaemia 33.3% Lymphomas 28.1% Solid tumours 38.6%	**Education and coaching** **(non-supervised)** *Duration of intervention:* n.a. *Frequency:* n.a. *Duration of a session:* n.a. *Intensity:* n.a.	Historical control group (usual care)
Keats and Culos-Read et al. (2008) [49]	SGI	**N = 10** age (mean): 16.2 ± 1.6 y Intensive cancer treatment Maintenance therapy After care	Leukaemia 40.0% Lymphomas 40.0% CNS tumours 10.0% Germ cell tumours 10.0%	**Multimodal training (supervised)** *Duration of intervention:* 16 Weeks *Frequency:* 1×/Week *Duration of a session:* 90 min *Intensity:* individually adapted	No CG
Khoirunnisa et al. (2019) [42]	QET	**N = 64** Intensive cancer treatment Maintenance therapy *IG (n = *32*):* age (mean): 11.69 ± 2.58 y *CG (n = *32*):* age (mean): 12.97 ± 3.20 y	Leukaemia and lymphomas 57.8% Solid tumours 42.2%	**AeRop (endurance training and PMR) (partly supervised)** *Duration of intervention:* 5 Days *Frequency:* daily *Duration of a session:* 30 min *Intensity:* n.a.	Usual care
Lam et al. (2018) [37]	RCT	**N = 70** Intensive cancer treatment *IG (n = *37*):* age (mean): 12.8 ± 2.5 y *CG (n = *33*):* age (mean): 12.97 ± 3.20 y	Leukaemia 42.9% Lymphomas 21.4% CNS tumours 15.7% Germ cell tumours 8.6% Bone tumours 5.7% Other solid tumours 5.7%	**Multimodal training (supervised)** *Duration of intervention:* 6 months *Frequency:* 1–2×/Week *Duration of a session:* 60 min *Intensity:* low–moderate	Placebo intervention (home visits with leisure activities)
Ovans et al. (2018) [50]	SGI	**N = 15** age (mean): 11.47 ± 3.33 y Intensive cancer treatment Maintenance therapy After care	CNS tumours	**Walking interventions withfitness tracker and coaching(non-supervised)** *Duration of intervention:* 2 Weeks *Frequency:* daily *Duration of a session:* n.a. *Intensity:* n.a.	No CG
Platschek et al. (2017) [51]	SGI	**N = 9** age (mean): 11.33 ± 2.24 y Intensive cancer treatment Maintenance therapy	Leukaemia 33.3% Lymphomas 33.3% Sarcomas 22.2% Neuroblastomas 11.1%	**Technology-based exercise** **intervention (supervised)** *Duration of intervention:* 12 Weeks *Frequency:* n.a. *Duration of a session:* 45 min *Intensity:* individually adapted	No CG
Rosenhagen et al. (2011) [43]	QET	**N = 23** age (mean): 15.3 ± 3.7 y Intensive cancer treatment *IG (n = *13*):* age: n.a. *CG (n = *10*):* age (mean): 13.6 ± 4.0 y	Leukaemia 79.0% Lymphomas 4.2% Neuroblastomas 4.2% Soft tissue sarcomas 4.2% Germ cell tumours 4.2% Haematol. disorders 4.2%	**Multimodal training (supervised)** *Duration of intervention:* 34.1 ± 9.4 days *Frequency:* 3×/Week *Duration of a session:* 50 min *Intensity:* moderate	Retrospective CG (usual care)
Spreafico et al. (2021) [44]	QET	**N = 44** age (median): 15.5 y Intensive cancer treatment Maintenance therapy After care *IG (n = *21*):* age: n.a. *CG (n = *23*):* age: n.a.	Bone tumours 31.8% CNS tumours 18.2% Soft tissue sarcomas 18.2% Lymphomas 11.4% Neuroblastomas 11.4% Nephroblastomas 4.6% Other solid tumours 4.6%	**Multimodal training (supervised)** *Duration of intervention:* 6 Weeks *Frequency:* 3×/Week *Duration of a session:* 60 min *Intensity:* moderate	Usual care
Stössel et al. (2020) [38]	RCT	**N = 33** Intensive cancer treatment *IG (n = *16*):* age (mean): 10.6 ± 5.19 y *CG (n = *17*):* age (mean): 11.4 ± 4.25 y	Leukaemia and lymphomas 45.5% CNS tumours 12.1% Other solid tumours 42.4%	**Multimodal training** **(partly supervised)** *Duration of intervention:* 6–8 Weeks *Frequency:* 3×/Week *Duration of a session:* 45–60 min *Intensity:* moderate	Usual care
Su et al. (2018) [52]	SGI	**N = 18** age (mean): 11.89 ± 4.63 y Intensive cancer treatment Maintenance therapy After care	Leukaemia 66.7% Solid tumours 33.3%	**Walking intervention with fitness tracker and education** **(partly supervised)** *Duration of intervention:* 6 Weeks *Frequency:* 5×/Week *Duration of a session:* 15–30 min *Intensity:* n.a.	No CG
van Dijk-Lokkart et al. (2016) [39]	RCT	**N = 68** Intensive cancer treatment Maintenance therapy After care *IG (n = *30*):* age (mean): 13.0 ± 3.0 y *CG (n = *10*):* age (mean): 12.6 ± 3.1 y	Leukaemia and lymphomas 67.7% Solid tumours 22.1% CNS tumours 10.3%	**Endurance and strength training** **and psychosocial training** **(supervised)** *Duration of intervention:* 12 Weeks *Frequency:* 2×/Week *Duration of a session:* 45 min *Intensity:* vigorous	Usual care
Yeh et al. (2011) [45]	QET	**N = 22** Intensive cancer treatment Maintenance therapy *IG (n = *12*):* age (mean): 11.01 ± 3.56 y *CG (n = *10*):* age (mean): 12.48 ± 3.86 y	Leukaemia	**Technology-based physical** **activity intervention** **(non-supervised)** *Duration of intervention:* 6 Weeks *Frequency:* 3×/Week *Duration of a session:* 30 min *Intensity:* low–moderate	Usual care

CG, control group; f., female; IG, intervention group; N, number; n.a., not available; QET, quasi-experimental trial; RCT, randomised controlled trial; SGI, single-group intervention study; y, years.

**Table 2:** Characteristics of the study population.

Characteristics	Number of Studies
Diagnosis	
Leukaemias	3
CNS tumours	1
Heterogenous entitities	16
Treatment	
CTx	9
CTx and RT	4
SCT	2
CTx, RT and surgery	1
CTX, RT and SCT	1
CTx and SCT	1
CTx and surgery	1
Unclear	1
Treatment phase	
Intensive cancer treatment	8
Maintenance therapy	1
Intensive cancer treatment and maintenance therapy	4
Maintenance therapy and after care	2
During all treatment phases	5

CTx, chemotherapy; RT, radiotherapy; SCT, stem-cell transplant.

**Table 3:** Methodological quality of included RCT.

	Criteria														
Author (Year)	1	2	3	4	5	6	7	8	9	10	11	12	13	Score	%
Atkinson et al. (2021) [33]	y	y	Y	u	u	y	u	n	u	y	y	n	y	7	53.85
Bhatt et al. (2013) [34]	y	y	U	u	u	u	n	u	u	y	y	u	y	5	38.46
Hamari et al. (2019) [35]	y	u	Y	y	y	n	y	y	n	y	u	y	y	9	69.23
Hinds et al. (2007) [36]	y	u	Y	n	n	n	u	u	y	y	y	n	y	6	46.15
Lam et al. (2018) [37]	y	y	Y	n	n	y	n	y	y	y	u	y	y	9	69.23
Stössel et al. (2020) [38]	y	y	Y	n	n	n	y	y	y	y	y	u	y	9	69.23
van Dijk-Lokkart et al. (2016) [39]	y	u	Y	u	u	u	y	y	u	y	y	y	y	8	61.54

Criteria: 1. Random sequence generation, 2. Allocation concealment, 3. Comparability of treatment groups, 4. Blinding of participants, 5. Blinding of study personnel, 6. Blinding of outcome assessment, 7. Comparability of treatment between treatment groups, 8. Completeness of follow-up, 9. Analysis of participants in randomly allocated groups, 10. Uniform measurement of results for all groups, 11. Reliable outcome measures, 12. Use of adequate statistical analysis, 13. Use of adequate study design and discussion of deviations; y = yes; n = no; u = unclear.

**Table 4:** Methodological quality of included QET and SGI.

	Criteria										
Author (Year)	1	2	3	4	5	6	7	8	9	Score	%
Bogg et al. (2015) [46]	y	Y	u	n	n	y	na	u	u	3	33.33
Däggelmann et al. (2017) [40]	y	N	n	y	y	u	y	y	u	5	55.56
Diorio et al. (2015) [47]	y	Y	n	n	y	u	na	y	n	4	44.44
Hooke et al. (2016) [48]	y	Y	y	n	y	u	na	y	u	5	55.56
Hooke et al. (2019) [41]	y	Y	u	y	n	n	n	y	u	4	44.44
Keats and Culos-Reed (2008) [49]	y	Y	n	n	y	y	na	u	n	4	44.44
Khoirunnisa et al. (2019) [42]	y	Y	y	y	y	u	y	y	u	7	77.78
Ovans et al. (2018) [50]	y	Y	n	n	y	y	na	y	u	5	55.56
Platschek et al. (2017) [51]	y	Y	y	n	y	y	na	y	u	6	66.67
Rosenhagen et al. (2011) [43]	y	N	n	n	y	u	y	y	u	4	44.44
Spreafico et al. (2021) [44]	y	N	y	y	n	na	n	y	u	4	44.44
Su et al. (2018) [52]	y	Y	n	n	y	y	na	u	u	4	44.44
Yeh et al. (2011) [45]	y	Y	y	y	y	y	y	u	u	7	77.78

Criteria: 1. Clearness about cause and effect, 2. Comparability of study participants, 3. Comparability of treatment between participants, 4. Availability of a control group, 5. Multiple outcome measures (pre/post-test), 6. Completeness of follow-up, 7. Uniform measurement of results for all groups, 8. Reliable outcome measures, 9. Use of adequate statistical analysis; y = yes; n = no; u = unclear; na = not applicable.

**Table 5:** Effects of physical activity interventions.

Author (Year)	Measurement of CRF	ADH	Within-Group Differences (IG)		Between-Group Differences	
			Post-Test	Follow-Up Test	Post-Test	Follow-Up Test
Atkinson et al. (2021) [33]	FS-C FS-A	90%	**Post-test** **(after 10 weeks):** CRF ↓ Endurance (VO_2peak_) ↑ Strength ↑ Flexibility ↑ QoL ↑ PA ↑ BMI ∼	**Follow-up** **(after 6 months):** CRF ↓ Endurance (VO_2peak_) ∼ Strength ↑ Flexibility ↑ QoL ↑ PA ↑ BMI ↑↑	**Post-test** **(after 10 weeks):** CRF ∼ Endurance (VO_2peak_) ++ Strength ∼ Flexibility ∼ QoL ∼ PA ∼ BMI ∼	**Follow-up** **(after 6 months):** CRF ∼ Endurance (VO_2peak_) ∼ Strength ∼ Flexibility ∼ QoL ∼ PA ∼ BMI ++
Bhatt et al. (2013) [34]	PFS	n.a.	**Post-test** **(after 3 weeks):** CRF ↓↓		**Post-test** **(after 3 weeks):** CRF- -	
Bogg et al. (2015) [46]	PedsQL MFS	63%	**Post-test** **(6 weeks after Tx):** CRF ∼ QoL ∼ Endurance (6MWT) ↓↓ Strength ∼ Balance ↓↓			
Däggelmann et al. (2017) [40]	PedsQL MFS	n.a.	**Post-test** **(after 4 weeks):** Total CRF ↓↓ General fatigue ↓↓ Cognitive fatigue ↓ Sleep/rest fatigue ↓↓ HrQoL ↑ Motor performance ↑	**Follow-up** **(after 7 months):** Total CRF ↓↓ General fatigue ↓↓ Cognitive fatigue ↓ Sleep/rest fatigue ↓↓ HrQoL ↑↑ Motor performance ↑↑		
Diorio et al. (2015) [47]	PedsQL MFS FS-C FS-A FS-P	n.a.	**Post-test** **(after 3 weeks):** General fatigue ∼ Sleep/rest fatigue ↑ CRF (parent-report) ↓ QoL ↑			
Hamari et al. (2019) [35]	PedsQL MFS	77%	**Post-test (after 8 weeks):** CRF ∼ PA ↓ Motor performance ↓		**Post-test (after 8 weeks):** CRF ∼ Step count + PA ∼ Motor performance ∼	**Follow-up (after 1 year):** Step count - -
Hinds et al. (2007) [36]	FS-Child FS-A FS-P FS-S	85%	**Post-test** **(daily for 3 days):** CRF (self-report) ∼ CRF (parent-report) ∼ CRF (staff-report) ∼ Sleep duration ∼		**Post-test** **(daily for 3 days):** CRF (self-report)- CRF (parent-report) + CRF (staff-report) + Sleep efficacy + Sleep duration +	
			*Significant negative correlation between CRF and sleep quality*			
Hooke et al. (2016) [48]	FS-C FS-A	92%	**Post-test** **(after 2 weeks):** CRF ∼ Step count ↑			
			*Significant negative correlation between step count and fatigue*			
Hooke et al. (2019) [41]	FS-C FS-A	n.a.	**Post-test** **(after 6 months):** All diagnosis groups: CRF↓ PA (self-report) ∼ Step count ∼ Sedentary time ∼ Solid tumours: CRF ↑↑ PA (self-report) ↓ Step count ∼ Sedentary time ∼	ALL: CRF↓ PA (self-report) ↑ Step count↓ Sedentary time ∼ Lymphomas: CRF↓ PA (self-report)↓ Step count ↑↑ Sedentary time ∼	**Post-test** **(after 6 months):** Differences between solid tumours and ALL/ lymphomas: CRF: *significantly higher in solid tumours* PA (self-report): *non-significantly lower in solid tumours*	
			*No correlation between PA and CRF*			
Keats and Culos-Read et al. (2008) [49]	PedsQL MFS	82%	**Post-test** **(after 16 weeks):** Total CRF ↓ General fatigue ↓↓ Cognitive fatigue ∼ Sleep/rest fatigue ↓ QoL ↑↑ PA ↑ Upper body strength ↑↑ Flexibility ↑↑ BMI ∼	**Follow-up** **(after 7 months):** Total CRF ↓↓ General fatigue ↓↓ Cognitive fatigue ↓ Sleep/rest fatigue ↓↓ QoL ↑↑ PA ↑↑ Upper body strength ↑↑ Flexibility ↑ BMI ↑		
Khoirunnisa et al. (2019) [42]	CFS	n.a.			**Post-test (at day 5):** CRF ++ Sleep quality ++	
Lam et al. (2018) [37]	FS-C FS-A	n.a.		**Follow-up** **(after 9 months):** CRF ↓↓ QoL ↑↑ PA ↑↑ Self-efficacy ↑↑ Hand-grip strength ↑↑	**Post-test** **(after 6 months):** CRF ++ QoL + Self-efficacy ++ Hand-grip strength ++	**Follow-up** **(after 9 months):** CRF ++ QoL ++ PA ++ Self-efficacy + Hand-grip strength +
Ovans et al. (2018) [50]	PedsQL MFS	n.a.	**Post-test** **(after 12 weeks):** Total CRF ↓↓ General fatigue ↓↓ Cognitive fatigue ↓ Sleep/rest fatigue ↓↓ QoL ↑ Step count ↑ Endurance (6MWT) ↑↑ PA ↑	**Follow-up** **(after 24 weeks):** Total CRF ↓↓ General fatigue ↓↓ Cognitive fatigue ↓ Sleep/rest fatigue ↓ QoL ↑ Step count ↑ Endurance (6MWT) ↑ PA ↑		
			*Significant negative correlation between CRF and step count*			
Platschek et al. (2017) [51]	PedsQL MFS	n.a.	**Post-test** **(after 12 weeks):** Total CRF ↓↓ General fatigue ↓ Cognitive fatigue ↓ Sleep/rest fatigue ↓↓ Physical condition ↑↑ Mental resilience ↑↑ Motivation ↑↑			
Rosenhagen et al. (2011) [43]	PedsQL MFS	n.a.	**Post-test** **(at discharge after Tx):** CRF ↓ QoL ↑ Hand-grip strength ↑ Endurance ∼			
Spreafico et al. (2021) [44]	PedsQL MFS	n.a.			**Post-test** **(after 6 weeks):** Total CRF- General fatigue + Cognitive fatigue - Sleep/rest fatigue - QoL +	
Stössel et al. (2020) [38]	PedsQL MFS	n.a.	**Post-test** **(after 6–8 weeks):** CRF (self report) ↓↓ CRF (parent report) ↓↓ QoL (self report) ↑ QoL (parent report) ∼ Leg strength ↑↑ Arm strength ↑ Endurance (6MWT) ↑↑ Phase angle ∼ BMI ∼ PA ↑↑		**Post-test** **(after 6–8 weeks):** CRF (self report) + CRF (parent report) ++ QoL (self report) + QoL (parent report) ∼ Leg strength ++ Arm strength ∼ Endurance (6MWT) ++ Phase angle ∼ BMI ∼ PA ++	
			*Significant correlation between PA and better physical and mental well-being, higher self-confidence and lower CRF values*			
Su et al. (2018) [52]	PedsQL MFS	72–89%	**Post-test** **(after 6 weeks):** Total CRF ↓↓ General fatigue ↓↓ Cognitive fatigue ↓↓ Sleep/rest fatigue ↓ QoL ↑ Endurance (6MWT) ↑↑ Sleep quality ∼ PA ↑↑			
van Dijk- Lokkart et al. (2016) [39]	PedsQL MFS	n.a.	**Post-test** **(after 4 months):** CRF (self report) ↓ CRF (parent report) ↓	**Follow-up** **(after 12 months):** CRF (self report) ↓ CRF (parent report) ↓	**Post-test** **(after 4 months):** CRF (self report) ∼ CRF (parent report) ∼ QoL ∼ Behav. disorders ∼ Depression ∼ Self-perception ∼	**Follow-up** **(after 12 months):** CRF (self report) ∼ CRF (parent report) ∼ QoL ∼ Behav. disorders ∼ Depression ∼ Self-perception ∼
Yeh et al. (2011) [45]	PedsQL MFS	ITT: 76% PP: 90%	**Post-test** **(after 6 weeks):** ITT: General fatigue ↓ Cognitive fatigue ↓ Sleep/rest fatigue ↓ PP: General fatigue ↓ Cognitive fatigue ↓ Sleep/rest fatigue ↓	**Follow-up** **(after 10 weeks):** ITT: General fatigue ↓ Cognitive fatigue ↓ Sleep/rest fatigue ↓ PP: General fatigue ↓ Cognitive fatigue ↓ Sleep/rest fatigue ↓	**Post-test** **(after 6 weeks):** ITT: General fatigue ∼ Cognitive fatigue + Sleep/rest fatigue + PP: General fatigue ∼ Cognitive fatigue + Sleep/rest fatigue +	**Follow-up** **(after 10 weeks):** ITT: General fatigue + Cognitive fatigue + Sleep/rest fatigue + PP: General fatigue ++ Cognitive fatigue + Sleep/rest fatigue +
			*No correlation between haemoglobin level and fatigue*			

++, significant advantage in intervention group compared to control group; +, tendential advantage without significant difference in intervention group versus control group; ∼, no differences; -, tendential disadvantage without significant difference in intervention group versus control group; - - significant disadvantage in intervention group compared to control group; ↑↑, significant increase; ↑, tendential increase; ↓↓, significant reduction; ↓, tendential reduction; 6MWT, 6 min walking test; ADH, adherence; CFS, Childhood Fatigue Scale; FACIT-F, Functional Assessment of Chronic Illness Therapy–Fatigue; FS-C/A/P/S, Fatigue Scale–Child/Adolsecent/Parent/Staff; IG, intervention group; ITT, intention-to-treat; PA, physical activity; PedsQL MFS, Pediatric Quality of Life Inventory-Multidimensional Fatigue Scale; PP, per-protocol.

## Data Availability

Not applicable.

## Supplementary Materials

The following supporting information can be downloaded at: https://www.mdpi.com/article/10.3390/children10030572/s1, Table S1: The detailed search strategy employed in each electronic database; Table S2: JBI Critical Appraisal Checklist [29]; Table S3: Methodological quality assessment of the included studies; Table S4: PRISMA Checklist [65]; Table S5: Deviations from initial PROSPERO statements.
