# Supplementary material for: Physical Activity as a Treatment for Cancer-Related Fatigue in Children, Adolescents and Young Adults: A Systematic Review
Source: Children (Basel). 2023 Mar 17;10(3):572. doi: 10.3390/children10030572 (PMC10047895; doi:10.3390/children10030572)
Supplement: Supplementary file 1 [file children-10-00572-s001.zip › S1_Search strategy.pdf]

S1: Search strategy

Search strategy in PubMed (MEDLINE)

("fatiguability"[All Fields] OR "fatiguable"[All Fields] OR "fatigue"[MeSH Terms] OR "fatigue"[All Fields] OR "fatigued"[All Fields] OR "fatigues"[All Fields] OR "fatiguing"[All Fields] OR "fatigueability"[All Fields]) AND ((("paediatrics"[All Fields] OR "pediatrics"[MeSH Terms] OR "pediatrics"[All Fields] OR "paediatric"[All Fields] OR "pediatric"[All Fields]) AND ("neoplasms"[MeSH Terms] OR "neoplasms"[All Fields] OR "oncology"[All Fields] OR "oncology s"[All Fields])) OR (("child"[MeSH Terms] OR "child"[All Fields] OR "children"[All Fields] OR "child s"[All Fields] OR "children s"[All Fields] OR "childrens"[All Fields] OR "childs"[All Fields]) AND ("neoplasms"[MeSH Terms] OR "neoplasms"[All Fields] OR "oncology"[All Fields] OR "oncology s"[All Fields])) OR (("adolescences"[All Fields] OR "adolescency"[All Fields] OR "adolescent"[MeSH Terms] OR "adolescent"[All Fields] OR "adolescence"[All Fields] OR "adolescents"[All Fields] OR "adolescent s"[All Fields]) AND ("neoplasms"[MeSH Terms] OR "neoplasms"[All Fields] OR "oncology"[All Fields] OR "oncology s"[All Fields]))) AND ((("exercise"[MeSH Terms] OR "exercise"[All Fields] OR "exercises"[All Fields] OR "exercise therapy"[MeSH Terms] OR ("exercise"[All Fields] AND "therapy"[All Fields]) OR "exercise therapy"[All Fields] OR "exercise s"[All Fields] OR "exercised"[All Fields] OR "exerciser"[All Fields] OR "exercisers"[All Fields] OR "exercising"[All Fields]) AND ("neoplasms"[MeSH Terms] OR "neoplasms"[All Fields] OR "oncology"[All Fields] OR "oncology s"[All Fields])) OR (("sport s"[All Fields] OR "sports"[MeSH Terms] OR "sports"[All Fields] OR "sport"[All Fields] OR "sporting"[All Fields]) AND ("neoplasms"[MeSH Terms] OR "neoplasms"[All Fields] OR "oncology"[All Fields] OR "oncology s"[All Fields])))

Search strategy in SPORTDiscus (EBSCO)

(fatigue) AND (pediatric oncology OR child oncology OR adolescent oncology) AND (exercise oncology OR sport oncology)<br><br>Interface - EBSCOhost Research Databases<br><br>Search Screen - Advanced Search<br><br>Database - RILM Music Encyclopedias; America: History & Life; American Antiquarian Society (AAS) Historical Periodicals Collection: Series 3;American Antiquarian Society (AAS) Historical Periodicals Collection: Series 1;American Antiquarian Society (AAS) Historical Periodicals Collection: Series 2;American Antiquarian Society (AAS) Historical Periodicals Collection: Series 4;American Antiquarian Society (AAS) Historical Periodicals Collection: Series 5;OpenDissertations;Business Source Premier; Communication Abstracts; eBook Collection (EBSCOhost); EconLit; GeoRef; GeoRef In Process; GreenFILE;Historical Abstracts; Index Islamicus; Index to Legal Periodicals and Books (H.W. Wilson); Library, Information Science & Technology Abstracts; MLA Directory of Periodicals; MLA International Bibliography; Philosopher's Index; PSYNDEX Literature with PSYNDEX Tests; Regional Business News; The Nation Archive (DFG);The New Republic Archive (DFG); RIPM - Retrospective Index to Music Periodicals; CINAHL Complete; APA PsycArticles; MEDLINE;APA PsycInfo; RILM Abstracts of Music Literature; eBook Subscription Psychology Collection (Trial);eBook Open Access (OA) Collection (EBSCOhost)<br><br>Expanders - Apply related words; Also search within the full text of the articles<br><br>Search modes - Boolean/Phrase
