# Supplementary material for: Physical Activity as a Treatment for Cancer-Related Fatigue in Children, Adolescents and Young Adults: A Systematic Review
Source: Children (Basel). 2023 Mar 17;10(3):572. doi: 10.3390/children10030572 (PMC10047895; doi:10.3390/children10030572)
Supplement: Supplementary file 1 [file children-10-00572-s001.zip › S2_JBI Critical Appraisal Checklist.pdf]

S2: JBI Critical Appraisal Checklist

Table S2.1 JBI Critical Appraisal Checklist for Randomized Controlled Trials (Tufanaru et al., 2020).

Item		Yes	No	Unclear	NA
**1**	Was true randomization used for assignment of participants to treatment groups?	☐	☐	☐	☐
**2**	Was allocation to treatment groups concealed?	☐	☐	☐	☐
**3**	Were treatment groups similar at the baseline?	☐	☐	☐	☐
**4**	Were participants blind to treatment assignment?	☐	☐	☐	☐
**5**	Were those delivering treatment blind to treatment assignment?	☐	☐	☐	☐
**6**	Were outcomes assessors blind to treatment assignment?	☐	☐	☐	☐
**7**	Were treatment groups treated identically other than the intervention of interest?	☐	☐	☐	☐
**8**	Was follow up complete and if not, were differences between groups in terms of their follow up adequately described and analyzed?	☐	☐	☐	☐
**9**	Were participants analyzed in the groups to which they were randomized?	☐	☐	☐	☐
**10**	Were outcomes measured in the same way for treatment groups?	☐	☐	☐	☐
**11**	Were outcomes measured in a reliable way?	☐	☐	☐	☐
**12**	Was appropriate statistical analysis used?	☐	☐	☐	☐
**13**	Was the trial design appropriate, and any deviations from the standard RCT design (individual randomization, parallel groups) accounted for in the conduct and analysis of the trial?	☐	☐	☐	☐

NA: Not applicable.

Table S2.2 JBI Critical Appraisal Checklist for Quasi-Experimental Studies (Tufanaru et al., 2020).

**Item**		**Yes**	**No**	**Unclear**	**NA**
**1**	Is it clear in the study what is the 'cause' and what is the 'effect' (i.e. there is no confusion about which variable comes first)?	☐	☐	☐	☐
**2**	Were the participants included in any comparisons similar?	☐	☐	☐	☐
**3**	Were the participants included in any comparisons receiving similar treatment/care, other than the exposure or intervention of interest?	☐	☐	☐	☐
**4**	Was there a control group?	☐	☐	☐	☐
**5**	Were there multiple measurements of the outcome both pre and post the intervention/exposure?	☐	☐	☐	☐
**6**	Was follow up complete and if not, were differences between groups in terms of their follow up adequately described and analyzed?	☐	☐	☐	☐
**7**	Were the outcomes of participants included in any comparisons measured in the same way?	☐	☐	☐	☐
**8**	Were outcomes measured in a reliable way?	☐	☐	☐	☐
**9**	Was appropriate statistical analysis used?	☐	☐	☐	☐

NA: Not applicable.
