# Supplementary material for: Physical Activity as a Treatment for Cancer-Related Fatigue in Children, Adolescents and Young Adults: A Systematic Review
Source: Children (Basel). 2023 Mar 17;10(3):572. doi: 10.3390/children10030572 (PMC10047895; doi:10.3390/children10030572)
Supplement: Supplementary file 1 [file children-10-00572-s001.zip › S3_Methodological quality assessment of the included studies.pdf]

S3: Methodological quality assessment of the included studies

Table S3.1: Methodological quality assessment of the included randomised controlled trials.

Autor (Jahr)	Item	Bewertung
Atkinson et al. (2021)	1.	Block randomisation with stratification factors
	2.	Concealed by opaque envelopes
	3.	No significant differences in baseline characteristics
	4.	No information about blinding of participants
	5.	No information about blinding of instructors
	6.	Outcome assessors were blinded
	7.	No information about distribution of different treatments in groups
	8.	More drop-outs in control arm; no description of impact
	9.	No information about ITT-analysis
	10.	Same measurements for all participants
	11.	Reliability of measurements is given
	12.	Linear mixed-effects model was performed; power calculation results were not respected; assumptions were met
	13.	Multicenter, parallel group randomized controlled trial
Bhatt et al. (2013)	1.	Randomisation using sealed envelope method
	2.	Concealed by opaque envelopes
	3.	No information about differences at baseline
	4.	No information about blinding of participants
	5.	No information about blinding of instructors
	6.	No information about blinding of outcome assessors
	7.	Different treatments in groups (IG 1: Aerobic exercise; IG 2: Relaxation techniques)
	8.	No information about drop-outs and missing data
	9.	No information about ITT-analysis
	10.	Same measurements for all participants
	11.	Reliability of measurements is given
	12.	Parametric tests were performed; no information about power calculation or assumption checks
	13.	Parallel group randomized controlled trial
Hamari et al. (2019)	1.	Computer-generated list based on block randomisation
	2.	No information about concealment
	3.	All characteristics seem to be comparable
	4.	Participants were blinded
	5.	Physical therapists were blinded
	6.	Outcome assessors were not blinded
	7.	No different treatments in groups
	8.	Comparable number of drop-outs in control arm; reasons for drop-outs are given
	9.	Missing data wasn't reported correctly
	10.	Same measurements for all participants
	11.	Incomplete information about reliability of measurements
	12.	Non-parametric tests were performed; power calculation results and assumptions were met
	13.	

		Parallel group randomized controlled trial with follow-up
Hinds et al. (2007)	1.	Computer-generated randomisation with stratification factors
	2.	No information about concealment
	3.	No significant differences in baseline characteristics
	4.	Participants were not blinded
	5.	Instructors were not blinded
	6.	Outcome assessors were not blinded
	7.	No information about different treatments in groups
	8.	Incomplete information about missing data and drop-outs
	9.	ITT-analysis was performed
	10.	Same measurements for all participants
	11.	Reliability of measurements is given
	12.	Parametric and nonparametric tests were performed; power calculation results were not respected; assumptions were met
	13.	Randomized, prospective, two-site and two-group pilot study
Lam et al. (2018)	1.	Computer-generated randomisation
	2.	Concealed by opaque envelopes
	3.	No significant differences in baseline characteristics
	4.	Participants were not blinded
	5.	Instructors were not blinded
	6.	Outcome assessors were blinded
	7.	Placebo intervention activities
	8.	Comparable number of drop-outs in control arm; reasons for drop-outs are given
	9.	ITT-analysis was performed
	10.	Same measurements for all participants
	11.	Incomplete information about reliability of measurements
	12.	Parametric and nonparametric tests were performed; power calculation results were respected
	13.	Two-group pre-test and repeated post-test between-subject design
Stössel et al. (2020)	1.	Computer-generated randomisation with stratification factors
	2.	Concealed randomisation with RITA software
	3.	No significant differences in baseline characteristics
	4.	Participants were not blinded
	5.	Instructors were not blinded
	6.	Outcome assessors were not blinded
	7.	Comparable treatments
	8.	Comparable number of drop-outs in control arm; reasons for drop-outs are given
	9.	ITT-analysis was performed
	10.	Same measurements for all participants
	11.	Reliability of measurements is given
	12.	Parametric test was performed; power calculation results were met; no information about assumption checks
	13.	Parallel group randomized controlled trial

Table S3.2 Methodological quality assessment of the included quasi-experimental trials and single-arm intervention trials.

**Autor (Jahr)**	**Item**	**Bewertung**
Bogg et al. (2015)	1.	Cause: Exercise intervention; Effect on: CRF
	2.	No CG; comparison between prepubertal children and adolescents
	3.	No CG
	4.	No CG
	5.	Pre- and post-Tx measurement; intervention began one week prior to Tx (no pretest)
	6.	Complete follow-up; reasons for missing data are given
	7.	No CG
	8.	Incomplete information about reliability of measurements
	9.	Parametric tests was performed; no information about power calculation or assumption checks
Däggelmann et al. (2017)	1.	Cause: Rehabilitation program; Effect on: CRF
	2.	IG: cancer children; CG: healthy siblings
	3.	No cancer treatment for healthy siblings; mediaction for cancer children
	4.	Healthy siblings
	5.	Measurements before and after the intervention and at follow-up
	6.	Incomplete follow-up; reasons for dropouts are given; no information about dropouts in specific group
	7.	Same outcome measures in groups
	8.	Reliability of measurements is given
	9.	Non-parametric tests were performed; no information about power calculations
Diorio et al. (2015)	1.	Cause: Individualized yoga; Effect on: CRF
	2.	No CG
	3.	No CG; different treatments for patients
	4.	No CG
	5.	Measurements at baseline, at day 7, 14 and 21
	6.	Incomplete follow-up; reasons for dropouts are given; no information about missing data
	7.	No CG
	8.	Reliability of measurements is given
	9.	No interferencial statistics were performed
Hooke et al. (2016)	1.	Cause: FitBit intervention; Effect on: CRF
	2.	No CG
	3.	No CG; Chemotherapy
	4.	No CG
	5.	Measurments before, during and after the intervention
	6.	Incomplete follow-up; reasons for dropouts are given; no information about missing data
	7.	No CG
	8.	Reliability of measurements is given
	9.	Nonparametric tests were performed; no information about power calculation

Hooke et al. (2019)	1. Cause: Education and Coaching (PA); Effect on: CRF<br>2. Comparable characteristics<br>3. No information about treatment of historical CG<br>4. Historical CG<br>5. No pretest; measurements in month 2, 4 and 6<br>6. Incomplete follow-up; no information about dropouts<br>7. Different measurement timepoints<br>8. Reliability of measurements is given<br>9. Parametric tests were performed; no information about power calculation or assumption checks
Keats und Culos-Reed (2008)	1. Cause: PA intervention; Effect on: CRF<br>2. No CG<br>3. No CG; Chemotherapy, Radiation, Both, Both + BMT<br>4. No CG<br>5. Measurements at baseline, after 8 and 16 weeks, 3-months postintervention,<br>6. 1-year poststudy initiation<br>7. Complete follow-up; reasons for missing data are given<br>8. No CG<br>9. Incomplete information about reliability of measurements<br>Parametric tests were performed; no information about power calculation and assumption checks
Khoirunnisa et al. (2019)	1. Cause: AeRop exercise intervention; Effect on: CRF<br>2. No significant differences between group characteristics<br>3. Comparable treatments<br>4. Standard care<br>5. Measurements at day 1 and day 5 after the intervention<br>6. No information about follow-up and dropouts<br>7. Same outcome measures in groups<br>8. Reliability of measurements is given<br>9. Parametric tests were performed; no information about power calculation or assumption checks
Ovans et al. (2018)	1. Cause: PA intervention; Effect on: CRF<br>2. No CG<br>3. No CG; sample included a variety of diagnoses, ages, cancer treatment regimens, and levels of comorbidities<br>4. No CG<br>5. Measurements at baseline, 12 and 24 weeks after the intervention<br>6. Incomplete follow-up; reasons for dropouts are given<br>7. No CG<br>8. Reliability of measurements is given<br>9. Parametric and nonparametric tests were performed; no information about power calculation
Platschek et al. (2017)	1. Cause: Computerbased exercise intervention; Effect on: CRF<br>2. No CG<br>3. No CG; chemotherapy<br>4. No CG<br>5. Measurements at baseline, 4, 8 and 12 weeks after the intervention

	6.	Complete follow-up; reasons for dropouts/missing data are given
	7.	No CG
	8.	Reliability of measurements is given
	9.	Parametric and nonparametric tests were performed; no information if assumption checks are respected
Rosenhagen et al. (2011)	1.	Cause: Exercise intervention; Effect on: CRF
	2.	CG: post-SCT (after care); IG: active treatment
	3.	breathing therapy, massage, mobilization therapy
	4.	Retrospective CG
	5.	Measurements at day 1 and 14, at discharge
	6.	Incomplete follow-up; reasons for dropouts are given; missing information about dropouts
	7.	Same outcome measures in groups
	8.	Reliability of measurements is given
	9.	No information about statistical procedure
Spreafico et al. (2021)	1.	Cause: Exercise intervention; Effect on: CRF
	2.	Significant differences between group characteristics
	3.	Comparable treatments
	4.	Standard care
	5.	IG: measurement after intervention; CG: measurement at baseline
	6.	No follow-up
	7.	Different measurement timepoints
	8.	Reliability of measurements is given
	9.	Parametric and nonparametric tests were performed;<br>no information about power calculation
Su et al. (2018)	1.	Cause: Walking intervention and education; Effect on: CRF
	2.	No CG
	3.	No CG; Active or completed treatment
	4.	No CG
	5.	Measurement before, during and after the intervention
	6.	Complete follow-up; reasons for dropouts/missing data are given
	7.	No CG
	8.	Incomplete information about reliability of measurements
	9.	Parametric tests were performed; no information about power calculation and assumption checks
Yeh et al. (2011)	1.	Cause: Home-based aerobic exercise intervention; Effect on: CRF
	2.	Comparable characteristics
	3.	Comparable treatment
	4.	Standard care
	5.	Measurement before, during and after the intervention; follow-up after 1 month
	6.	Incomplete follow-up; reasons for dropouts/missing data are given
	7.	Same outcome measures in groups
	8.	Incomplete information about reliability of measurements
	9.	Mixed-effects model was performed;no information about power calculation or assumption checks
