# Supplementary material for: Physical Activity as a Treatment for Cancer-Related Fatigue in Children, Adolescents and Young Adults: A Systematic Review
Source: Children (Basel). 2023 Mar 17;10(3):572. doi: 10.3390/children10030572 (PMC10047895; doi:10.3390/children10030572)
Supplement: Supplementary file 1 [file children-10-00572-s001.zip › S5_Deviations from PROSPERO.pdf]

S4: Deviations from indications given in PROSPERO

PROSPERO items	Deviation (Yes / No)	Description of deviation	Comments
**Review title**	Yes	The title in PROSPERO was a working title, which corresponds in content to the current title.	
**Authors**	No		
**Citation**	Yes	According to the change in the title the citation will slightly differ.	
**Review question**	No		
**Searches**	No		The search strategy was developed using terms and MeshTerms that combined the population ("oncology OR child oncology OR adolescent oncology"), the interventions ("exercise OR sport") and the outcome of interest ("fatigue").
**Types of study to be included**	No		
**Condition or domain being studied**	No		
**Participants/population**	Yes	At least 75% of the subjects were children, adolescents or young adults under 21 years of age.	To avoid the exclusion of studies reporting mainly on children, but also on a few young adult patients, it was necessary adapt the population (inclusion criteria) to a study population in which at least 75 percent of the subjects were children, adolescents or young adults under 21 years of age.
**Intervention(s), exposure(s)**	No		Physical activity interventions included any type of intervention in which skeletal muscles produced any bodily movement
**Comparator(s)/control**	No		
**Main outcome(s)**	No		
**Additional outcome(s)**	No		
**Data extraction (selection and coding)**	No		Described in a more detailed way in the manuscript.

**Risk of bias (quality) assessment**	No		Described in a more detailed way in the manuscript.
**Strategy for data synthesis**	Yes	No meta-analysis was conducted.	Since included studies have shown a large heterogeneity no meta-analysis was conducted.
**Analysis of subgroups or subsets**	Yes	Solely the impact of different physical activity interventions on CRF was investigated. No further analysis regarding the various impact of these interventions on populations with different cancer diagnosis was performed.	
**Contact details for further information**	No		
**Organisation affiliation of the review**	No		
**Review team members and their organisational affiliation**	No		
**Type and method of review**	Yes	No conduction of a meta-analysis	
**Funding sources/sponsors**	Yes	The project has also received funding from the Kinderkrebshilfe Mainz e.V. (next to the funding from the European Union's Horizon Project).	
**Conflicts of interest**	Yes	No conflicts of interest.	Nothing is currently stated in PROSPERO.
**Language**	Yes	The review will solely be published in English (and not also in German language).	
